# Impact of Teledermoscopy on Melanoma Diagnosis and Triage Efficiency in Northern Sweden

**DOI:** 10.2340/actadv.v106.44052

**Published:** 2026-02-10

**Authors:** Antonia LINDFORS, Virginia ZAZO, Daniel NOSEK, Per HALLBERG, Zinaida BUCHARBAJEVA, Nina LYCKSELL, Nirina ANDERSSON

**Affiliations:** 1 Department of Public Health and Clinical Medicine, Dermatology and Venereology, Umeå University, Umeå; 2 Department of Medical Biosciences, Umeå University, Umeå; 3 Department of Applied Physics and Electronics, Umeå University, Umeå, Sweden

**Keywords:** cutaneous melanoma, dermoscopy, malignant melanoma, skin cancer, teledermoscopy, telemedicine

## Abstract

The increasing incidence of cutaneous melanomas requires safe, efficient diagnostic methods to improve patient outcomes while optimizing healthcare resource utilization. Teledermoscopy was incorporated into clinical practice in 2014 in northern Sweden to improve diagnostic accuracy of cutaneous lesions and increase access to specialist dermatology care. This study aimed to evaluate whether teledermoscopy has led to earlier and more efficient diagnostics of cutaneous melanomas. Data on all excised pigmented cutaneous lesions from County Council of Västerbotten analysed at the Department of Clinical Pathology from 2010 to 2019 were collected. Before the introduction of teledermoscopy, the mean Breslow thickness was significantly higher (1.44 mm vs 1.08 mm, *p*=0.008), the proportion of Breslow ≤ 0.8 mm significantly lower (51.4% vs 70.6%, *p*< 0.001), and the mean Number Needed to Excise (23.32 vs 9.01, *p*< 0.001) significantly higher before the introduction of teledermoscopy. This indicates that teledermoscopy contributes to earlier and more efficient melanoma diagnosis.

## SIGNIFICANCE

Malignant melanoma is a serious form of cutaneous cancer, with potentially lethal outcome if not treated in time. Early diagnosis and treatment are crucial for prognosis and survival. Incorporating teledermoscopy into clinical practice, i.e., store-forward pictures enabling evaluation at a distance, has led to more efficient and earlier diagnosis of cutaneous melanomas. This ultimately leads to improved patient outcomes while optimizing healthcare resource utilization.

Cutaneous melanoma (CM) has one of the fastest rising incidence rates of all types of cancers, and the incidence of melanomas in Sweden is among the highest in Europe. Each year, approximately 500 patients die from CM in Sweden (1). Moreover, CM accounts for the single highest societal costs of all cutaneous cancers in Sweden (2). Therefore, safe and efficient management of CM is essential for optimizing the use of healthcare resources and improving patient outcomes.

Early detection and treatment with surgical excision are key to a good prognosis of CM. Unfortunately, benign cutaneous lesions are often unnecessarily excised, which consumes healthcare resources and exposes patients to potential risks such as emotional stress and infections. Diagnoses can also be delayed due to limited healthcare resources.

A commonly used measurement of diagnostic efficiency is the Number Needed to Excise (NNE), calculated by dividing the total number of excised cutaneous lesions (CL) by the number of CM, where a low value of NNE indicates a low rate of unnecessary excisions. Previous studies have shown that dermatology specialists have lower NNE values (3, 4) and shorter time until diagnosis and treatment of CM compared with general specialists (5). Thus, involving dermatology specialists in the triage and diagnostics of CM could lead to earlier and more efficient diagnosis of CM.

However, access to dermatology specialist care is limited, especially in the northern parts of Sweden. The region of Västerbotten has previously reported lower rates of early melanoma detection, where long travel distances as well as the large catchment area for the single regional specialist clinic could be contributing factors (6). In 2014, teledermoscopy (TD) was introduced in clinical practice as an attempt to increase access to dermatology specialist care by structural improvement. We have previously shown that TD in northern Sweden has high diagnostic accuracy (sensitivity 98.9%), as well as a high degree of early diagnostics (7).

In this study, we aimed to analyse whether the introduction of TD has led to earlier diagnosis of CM in northern Sweden. We also aimed to investigate whether TD has contributed to improved diagnostic efficiency of CM, with fewer unnecessary excisions.

## MATERIALS AND METHODS

In this retrospective observational study, data from all excised pigmented CL from the County Council of Västerbotten, analysed by the Department of Clinical Pathology at Umeå University Hospital, in northern Sweden, between 2010 and 2019 were collected. Since this Pathology Department is the only one in the County Council of Västerbotten, all of the pigmented CL from this geographical region are analysed there. During the study period the population size in the County of Västerbotten ranged between 259,286 in 2010, and 271,736 in 2019. The pigmented CLs were defined according to certain diagnosis codes, and the included samples were divided into 3 groups: benign, intermediate, and malignant diagnoses (**Table I**). Recurrences of CL were regarded as new lesions and were included in the study, while duplicates, non-cutaneous samples, metastases, and samples from other geographical regions were excluded. The year 2014, when TD was implemented in clinical practice, was excluded from further analyses as it was considered a learning period.

**Table I:** Included benign, intermediate, and malignant diagnoses

Diagnosis	Diagnosis code
Benign	
Blue nevus	M87800
Dermatofibroma	M88320
Lichenoid keratosis	M48892
Pigmented lesion UNS	M57100
Pigmented nevus	M87200, M87600, M87400, M87230, M75700
Seborrhoeic keratosis	M72750
Intermediate	
Atypical/dysplastic nevus	M87431, M87270
Atypical pigmented tumour	M87201
Melanocytoma	M87281
Spitz nevus/tumour	M87799, M87720, M87700
Malignant	
Acral lentiginous melanoma	M87443, M87453
*In situ*/lentigo maligna	M87202, M87422, M87452
Lentigo malignant melanoma	M87423
Malignant melanoma UNS	M87203
Nodular melanoma	M87213
Superficial spreading melanoma	M87433
Suspected malignant melanoma/MELTUMP/STUMP/SAMPUS	M69700

MELTUMP: melanocytic tumour of uncertain malignant potential; SAMPUS: superficial atypical melanocytic proliferation of uncertain significance; STUMP: Spitz tumour of uncertain malignant potential; UNS: unspecified.

Breslow thickness, Clark level, and presence or absence of ulceration were specified for each sample where it was available in the pathology statement. If multiple values of Breslow thickness were present for the same lesion (e.g., primary biopsy/excision and extended excision), the highest value was chosen. Unless otherwise stated by the pathologist, melanoma *in situ* and lentigo maligna were considered non-invasive and received a Breslow thickness of 0 mm, Clark level I, and absence of ulceration. Data were lacking for Breslow thickness in 48 cases (3.4%), Clark level in 69 cases (5.0%), and ulceration in 239 cases (17.1%) (**Table II**).

**Table II:** Characteristics of included malignant samples

Characteristics	Total	Women	Men
Samples, *n* (%)	1,394	726/1,394 (52.1)	668/1,394 (47.9)
Age at diagnosis, years, mean (SD)	64.3 (15.3)	62.7 (15.9)	66.0 (14.4)***
< 50 years, *n* (%)	259/1,394 (18.6)	158/726 (21.8)	101/668 (15.1)**
50–70 years, *n* (%)	608/1,394 (43.6)	324/726 (44.6)	284/668 (42.5)
> 70 years, *n* (%)	527/1,394 (37.8)	244/726 (33.6)	283/668 (42.4)***
Tumour site, *n* (%)			
Head/neck	132/1,394 (9.5)	65/726 (9.0)	67/668 (10.0)
Trunk	286/1,394 (20.5)	100/726 (13.8)	186/668 (27.8)***
Extremities	234/1,394 (16.8)	164/726 (22.6)	70/668 (10.5)***
Skin unspecified	742/1,394 (53.2)	397/726 (54.7)	345/668 (51.6)
Tumour type, *n* (%)			
MM UNS	293/1,394 (21.0)	139/726 (19.1)	154/668 (23.1)
SSM	336/1,394 (24.1)	191/726 (26.3)	145/668 (21.7)*
NM	130/1,394 (9.3)	56/726 (7.7)	74/668 (11.1)*
LMM	70/1,394 (5.9)	30/726 (4.1)	40/668 (6.0)
ALM	14/1,394 (1.0)	5/726 (0.7)	9/668 (1.3)
*In situ*/lentigo maligna	517/1394 (37.1)	282/726 (38.8)	235/668 (35.2)
Other	34/1394 (2.4)	23/726 (3.2)	11/668 (1.6)
Breslow, mm			
Mean (SD)	1.22 (2.20)	1.13 (2.16)	1.31 (2.23)
≤ 0.8, *n* (%)	855/1,346 (63.5)	460/693 (66.4)	395/653 (60.5)*
> 0.8, *n* (%)	491/1,346 (36.5)	233/693 (33.6)	258/653 (39.5)*
Clark level, *n* (%)			
I	513/1,325 (38.7)	279/685 (40.7)	234/640 (36.6)
II–III	456/1,325 (34.4)	233/685 (34.0)	223/640 (34.8)
IV–V	356/1,325 (26.9)	173/685 (25.3)	183/640 (28.6)
Ulceration, *n* (%)			
No	1,006/1,155 (87.1)	535/596 (89.8)	471/559 (84.3)**
Yes	149/1,155 (12.9)	61/596 (10.2)	88/559 (15.7)**

ALM: acral lentiginous melanoma; *In situ*: melanoma *in situ*; LMM: lentigo malignant melanoma; mm: millimetre; MM UNS: malignant melanoma unspecified; NM: nodular melanoma; Other: MELTUMP, SAMPUS, STUMP, Suspected malignant melanoma: SD, standard deviation; SSM: superficial spreading melanoma. Explanation of abbreviation 2.

Comparison between men and women (with independent samples *t*-test or χ^2^ test) =

* *p*< 0.05,

** *p*< 0.01,

*** *p*< 0.001.

### Statistical analysis

Statistical analyses were performed using SPSS Statistics for Windows, version 29.0 (IBM Corp, Armonk, NY, USA). For comparative analyses between 2 groups, the independent samples *t*-test was used for continuous data and the χ^2^ test was used for categorical data.

The mean Breslow thickness from 2010 to 2013 (before TD was introduced) was compared with the mean Breslow thickness from 2015 to 2019 (after TD was introduced). Additionally, the number of samples with a Breslow thickness of ≤ 0.8 mm was compared between the periods before and after the introduction of TD.

The yearly NNE values during the period 2010 to 2019 were calculated in 2 separate ways: by dividing the total number of excised CL by the number of excised malignant CL (NNE-M), and by dividing the total number of excised CL by the number of excised intermediate and malignant CL (NNE-IM). The mean NNE value from 2010 to 2013 (before TD was introduced) was compared with the mean NNE value from 2015 to 2019 (after TD was introduced). Furthermore, simple linear regression analyses were performed with NNE-M and NNE-IM as dependent variables.

Simple linear regression analyses with Breslow thickness as dependent variable, and sex, age at diagnosis, and year of diagnosis before or after TD as covariates, were also performed. The significant variables from simple linear regression were included in a multivariable linear regression model to adjust for potential confounders.

The study obtained ethical approval from the Regional Ethics Committee at the University Hospital in Umeå (Dnr: 2019–06165).

## RESULTS

A total of 15,005 benign, 2,475 intermediate, and 1,394 malignant samples were included in the study. The overall mean age at diagnosis for benign, intermediate, and malignant samples was 49.7 years (range 0–100 years), 48.2 years (range 1–92 years), and 64.3 years (range 15–99 years), respectively. The most common benign, intermediate, and malignant diagnoses were pigmented nevus (*n*=6,856), atypical/dysplastic nevus (*n*=2,306), and melanoma *in situ*/lentigo maligna (*n*=517), respectively.

There was a significant difference in mean age at diagnosis between men (66.0 years) and women (62.7 years) (*p*< 0.001). The trunk was the most common tumour site in men while the extremities were the most common localization in women (*p*< 0.001). The proportion of samples with Breslow thickness > 0.8 mm (39.5% vs 33.6%, *p*=0.025), and the presence of ulceration (15.7% vs 10.2%, *p*=0.005) were significantly higher in men (see Table II).

### Number of excisions

**Fig. 1:**
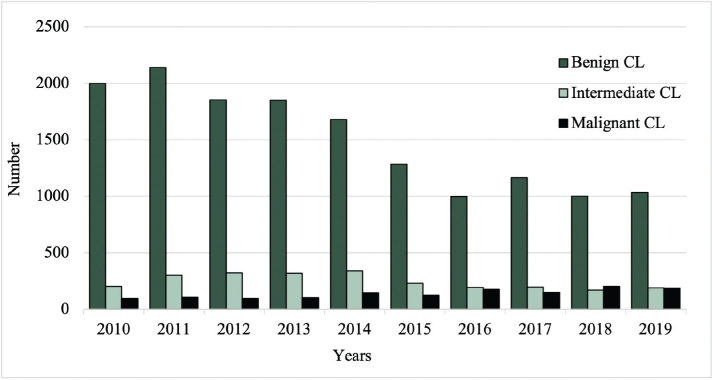
Total number of excisions of benign, intermediate, and malignant cutaneous lesions (CL) from 2010 to 2019.

**Fig. 2:**
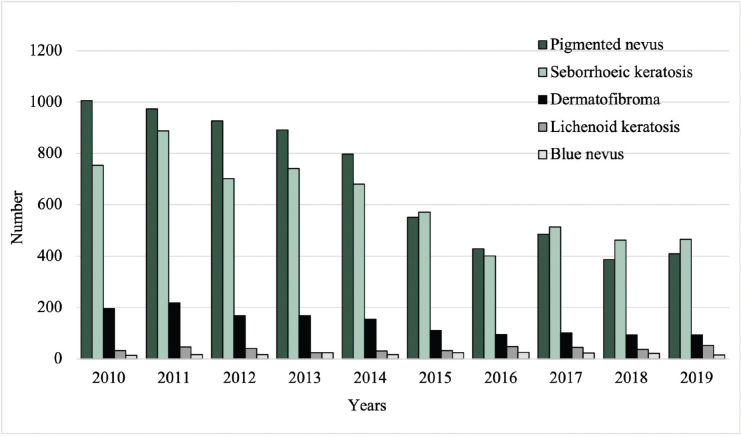
Excision rates of the most common benign cutaneous lesions from 2010 to 2019.

**Fig. 3:**
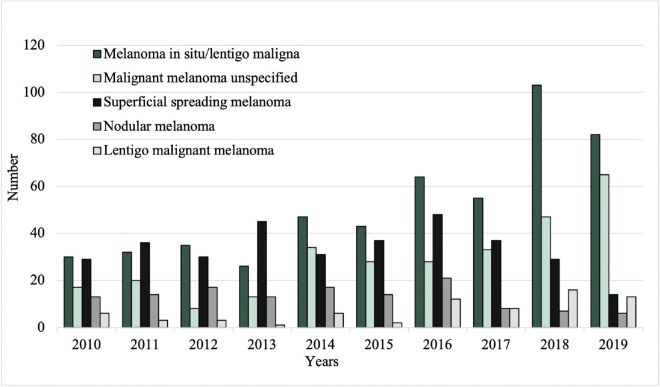
Excision rates of the most common malignant cutaneous lesions from 2010 to 2019.

Between 2010 and 2019, the total number of excised malignant CL per year increased from 96 to 187, while the number of excised benign CL decreased from 2,000 to 1,033 (**Fig. 1**). Among the most common benign lesions, the number of excisions decreased of pigmented nevus (1,006 to 409), seborrheic keratosis (754 to 466), and dermatofibroma (195 to 93) (**Fig. 2**). Of the most common malignant CL, the number of excised lesions increased for malignant melanoma unspecified (17 to 65), lentigo malignant melanoma (6 to 13), and melanoma in situ (30 to 82) (**Fig. 3**). However, there was a decrease of excised superficial spreading melanoma (29 to 14) and nodular melanoma (13 to 6) between 2010 and 2019 (Fig. 3).

### Number Needed to Excise (NNE)

**Fig. 4:**
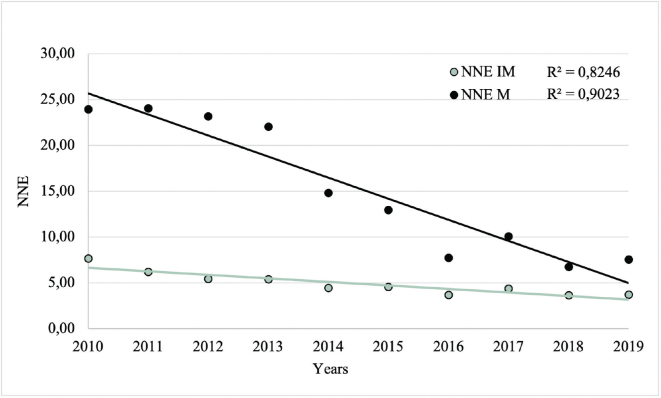
**Number Needed to Excision (NNE) values from 2010 to 2019, excluding (NNE-M) and including (NNE-IM) intermediate cutaneous lesions as necessary excisions.** NNE-M: total number of excised cutaneous lesions/excised malignant cutaneous lesions. NNE-IM: total number of excised cutaneous lesions/excised intermediate and malignant cutaneous lesions.

NNE, calculated both with excluding intermediate CL (NNE-M) and including intermediate CL (NNE-IM) as necessary excisions, decreased from 2010 to 2019. NNE-M decreased from 23.96 to 7.55 and NNE-IM decreased from 7.67 to 3.73 (**Fig. 4**). The overall mean NNE-M and NNE-IM were 15.32 and 4.92, respectively. The mean NNE-M (23.32 vs 9.01, *p*< 0.001) and NNE-IM (6.17 vs 4.00, *p*=0.004) were significantly higher before (2010–2013) compared with after (2015–2019) the introduction of TD.

In simple linear regression analyses, with NNE-M and NNE-IM as dependent variables, the year of diagnosis was significantly associated with both NNE values (*p*< 0.001 and *p*< 0.001, respectively).

### Breslow thickness, Clark level, and ulceration

**Fig. 5:**
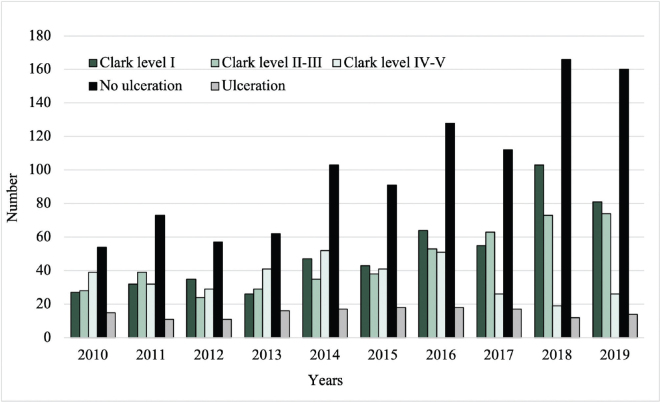
Clark level and frequency of ulceration of the malignant cutaneous lesions from 2010 to 2019.

Among the malignant CL, 63.5% had a Breslow thickness ≤ 0.8 mm, 38.7% Clark level I, and 87.1% absence of ulceration. The number of malignant CL with Clark level I and absence of ulceration increased from 27 to 81 and from 54 to 160, respectively, between 2010 and 2019 (**Fig. 5**).

**Fig. 6:**
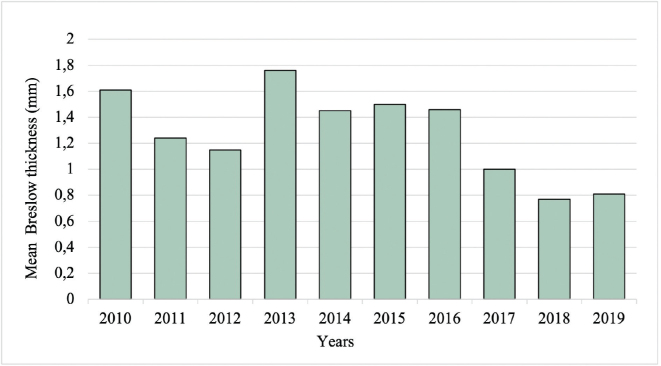
**Mean Breslow thickness of the malignant cutaneous lesions from 2010 to 2019.** Mm: millimetres.

The overall mean Breslow thickness was 1.22 mm, with a decrease from 1.61 mm in 2010 to 0.81 mm in 2019 (**Fig. 6**). The mean Breslow thickness was significantly higher (1.44 mm vs 1.08 mm, *p*=0.008) and the proportion of samples with Breslow thickness ≤ 0.8 mm was significantly lower (51.4% vs 70.6%, *p*< 0.001) before (2010–2013) compared with after (2015–2019) the introduction of TD.

In simple linear regression analyses, age at diagnosis and timing of diagnosis (before or after TD) were significantly associated with Breslow thickness (*p*=0.003 and *p*=0.008, respectively). After incorporating significant factors from the simple linear regression in a multivariable regression model to adjust for potential confounders, the association between timing of diagnosis and Breslow thickness remained statistically significant (*p*=0.004) (**Table III**).

**Table III:** Simple and multivariable linear regression with Breslow thickness as dependent variable, adjusted for age

Test variables	Simple			Multivariable		
	β-value	*p*-value	95% CI	β-value	*p*-value	95% CI
Sex	–0.18	0.123	–0.42–0.05	–		
Age at diagnosis	0.01	0.003	0.00–0.02	0.01	< 0.001	0.01–0.02
Diagnosis before or after TD	0.37	0.008	0.10–0.63	0.41	0.004	0.13–0.67

Diagnosis before teledermoscopy (TD): diagnosis between 2010 and 2013.

Diagnosis after TD: diagnosis between 2015 and 2019.

## DISCUSSION

In this study, we have shown that the implementation of TD has significantly improved the efficiency of CM triage in northern Sweden. Unnecessary excisions of benign lesions, calculated as NNE values, have decreased using TD, indicating a more efficient use of healthcare resources. Furthermore, Breslow thickness has decreased since the introduction of TD in clinical practice, indicating earlier diagnosis, and thus resulting in better prognoses and patient outcomes.

We found that both NNE-IM and NNE-M values significantly decreased after the implementation of TD. These findings are consistent with previous studies, which have observed a decrease in NNE values over time (4, 8). In those studies, however, the exact reasons for decreased NNE values were not determined, although improved diagnostic skills and incorporation of dermoscopy in clinical practice to a larger extent were suggested explanations. These factors may also contribute to the observed decrease in NNE values in the present study. However, we made comparisons before and after TD, which was not included in the other analyses. They also compared NNE values between specialized clinics vs non-specialized clinics, differences between the sexes, tumour site, and age at diagnosis. In some cases, CL display atypical features, making a clinical assessment of malignancy difficult, thus necessitating excision of the whole lesion for histopathological diagnosis. Excisions of intermediate CL were therefore not defined as unnecessary. To account for this, we calculated NNE in 2 ways: excluding intermediate CL (NNE-M) and including them (NNE-IM), to better reflect clinical complexity.

Our analyses showed that Breslow thickness was significantly lower following the introduction of TD. We also found that the majority of CM were thin (63.5% ≤ 0.8 mm), comparable to the proportion of thin CM (≤ 1 mm) presented in the Swedish Melanoma Registry in 2020 to 2023 (61% in men, and 63% in women) (1). Breslow thickness is one of the most important prognostic factors of CM. Given that tumours with a Breslow thickness of ≤ 0.8 mm are associated with a more favourable outcome (9,10), this threshold was used for analysis. In 2018, a specifically high increase in melanoma *in situ* was observed. Other explanatory factors for this increase could have been a shift in pathology assessment since a change in the WHO Classification of Skin Tumours was introduced in 2018 (11). These criteria could have affected the distinction between melanoma in situ and dysplastic nevi. However, in our calculations with NNE-IM, including intermediate lesions and dysplastic nevi, the results of improved diagnostic efficiency remained significant. We also considered that the implementation of TD in clinical practice may have required a learning period; therefore, the year when TD was implemented was excluded from the comparisons. However, this transition period may vary between clinics and may not be accurately represented by excluding a single year.

After the introduction of TD, the mortality rate in CM decreased to a larger extent in Västerbotten compared with the other counties in northern Sweden (Västernorrland, Jämtland, and Norrbotten) which had not yet implemented TD (12). The mortality rate in CM decreased from 6.2/100,000 inhabitants to 5.7/100,000 inhabitants in Västerbotten while in the other northern counties of Sweden the mortality did not improve to the same extent, or even increased. Before the introduction of TD, 13% of the CM in Västerbotten had a Breslow > 4 mm, which was higher than the national average, and a low proportion of thin CM, only 46.9% (6). However, after the implementation of TD, there was an increase in thin melanoma and a lower proportion of thick CM compared with the other regions in northern Sweden. These findings of earlier detection of CM with TD are in line with a previous Swedish cohort study by Schultz et al., reporting a higher proportion of *in situ* melanomas, and a significantly lower Breslow thickness in invasive melanomas (0.74 mm vs 1.35 mm, *p*=0.05) (13).

The number of excised malignant CL nearly doubled, while the number of excised benign CL nearly halved from 2010 to 2019. In concordance with these findings, the incidence of CM has steadily increased in Sweden and several other countries (1, 14–17). In contrast, a recent study by Helgadottir et al. has for the first time reported a decreasing incidence and mortality of CM in younger age groups in Sweden (18). This trend remains unclear, but possible explanations include changing population demographics, more efficient melanoma treatment, UV protection, and public health campaigns. Similar observations of decreasing incidence rates in younger people have been observed in Australia and the United States (19, 20).

Between men and women, significant differences were found concerning age at diagnosis, tumour site, Breslow thickness, and presence or absence of ulceration. In line with previous findings, men had a higher age at diagnosis, and the trunk was the most common localization for CL (1). In our study, men also had a higher proportion of nodular melanoma, Breslow thickness of > 0.8 mm, and presence of ulceration. In previous reports, male sex has been associated with a worse prognosis and is more likely to have more negative prognostic factors at the time of diagnosis (9, 15, 21, 22). This is not fully understood, although it might be explained by women seeking care earlier and being more likely to detect CM themselves (5, 23). Regarding the specific subtypes of CM, many were classified in this study as malignant melanoma unspecified (21.0%) rather than a specified subtype, contributing to difficulties in drawing any conclusions regarding changes in histological subtypes over time.

### Strengths and limitations

A major strength of the present study is the large sample size, including all specimens analysed at the Department of Pathology in the subsequent analyses. The study also benefits from access to detailed information for each case and a low proportion of missing data. Moreover, the long follow-up period of 9 years adds to the robustness of the study. All these factors enhance the reliability and generalizability of our findings.

Limitations of the study include the lack of adjustment for all potential confounders, such as the patients’ skin types. Additionally, we did not account for temporal changes in skin cancer awareness among patients and healthcare professionals, which may have influenced the likelihood of early CM detection. Also, data were not available to differentiate which of the included samples were diagnosed through TD vs traditional face-to-face consultations, limiting our ability to attribute outcomes exclusively to TD. We only have information regarding the proportion of melanomas evaluated via TD in 2019, which was 70%, although this likely varied between the years (7). However, the implementation of TD could potentially have improved access to traditional consultations as well, by optimizing healthcare resources, and thereby improved access and management in several ways.

### Conclusion

Both Breslow thickness and NNE values were significantly lower following the introduction of TD, suggesting that the introduction of TD has contributed to earlier diagnosis of CM and a reduction in unnecessary excisions. These findings support the integration of TD into clinical practice as a means of optimizing diagnostic precision and improving patient outcomes.
